# Cardiac Contractility Modulation: A Technical Review

**DOI:** 10.19102/icrm.2022.13102

**Published:** 2022-10-15

**Authors:** Aaron B. Hesselson

**Affiliations:** ^1^University of Kentucky Division of Cardiovascular Medicine, Lexington, KY, USA

**Keywords:** Cardiac contractility modulation, management, timing

## Abstract

Therapeutic options for the management of systolic congestive heart failure involving newer implantable device technologies that were not previously available now exist. Cardiac contractility modulation (CCM) has been shown to benefit patients with class III systolic heart failure with an ejection fraction of 25%–45% not indicated for biventricular pacing. Despite the increased use of CCM, there is a significant knowledge deficit in the general electrophysiology community regarding its clinical management. This significantly relates to the different functional characteristics of CCM compared to those encountered in traditional cardiac rhythm management devices. Improved evaluation and troubleshooting of CCM for therapeutic effect will benefit from advanced education. The goal of this review is to present pertinent technical aspects relating to CCM therapy management in a simplified fashion, all the while providing a clinical context to facilitate greater expertise.

## Introduction

Cardiac contractility modulation (CCM) therapy provides a non-excitatory stimulus to the heart that treats congestive heart failure by affecting improved myocardial cellular calcium handling and reversal of adverse gene dysregulations.^1–4^ Multicenter trials have demonstrated the clinical benefits of CCM for the management of medically refractory class III systolic heart failure not indicated for biventricular pacing therapy and with an ejection fraction of 25%–45%.^5–8^ Accordingly, the Optimizer Smart™ implantable pulse generator (IPG) (Impulse Dynamics, Marlton, NJ, USA) received expedited approval in March 2019 from the U.S. Food and Drug Administration (FDA) for these indications (Optimizer Smart™ System P180036).

Complicating the use of CCM is a considerable knowledge deficit amongst the electrophysiology community, physicians, nurses, and device specialists alike regarding the clinical management of CCM therapy. Additionally, a desire to minimize additional implanted endovascular hardware apart from a defibrillator or pacemaker has likely contributed to limiting the experience with CCM. This review describes the fundamental technical aspects of the Optimizer^®^ IPG and the Impulse Dynamics OMNI™II programmer pertinent to practical CCM therapy management to address this shortfall.

## Cardiac contractility modulation implanted hardware

**Figure 1:**
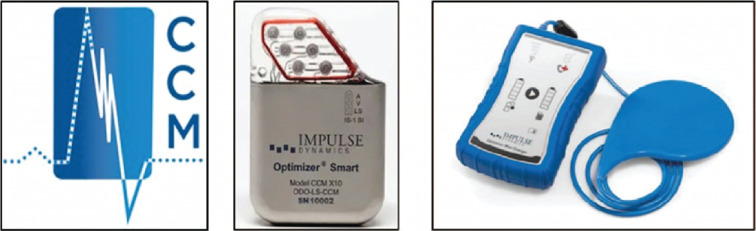
Optimizer Smart™ implantable pulse generator and Mini Charger™. Images courtesy of Impulse Dynamics.

**Figure 2:**
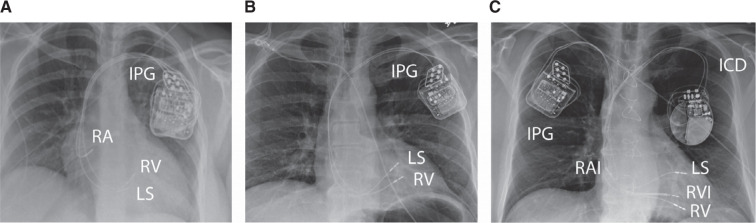
Chest radiograph of a cardiac contractility modulation (CCM) implant. Displayed are **(A)** a 3-lead CCM system only, **(B)** a 2-lead CCM system only, and **(C)** a 2-lead CCM system with a dual-chamber implantable cardioverter-defibrillator. Of note, the designation of the septal CCM leads as “LS” and “RV” is not determined by location, but rather the differential timing of sensing on each lead at implant. *Abbreviations:* CCM, cardiac contractility modulation; ICD, implantable cardioverter-defibrillator; IPG, implanted pulse generator; LS, local sense; RA, right atrial; RAI, right atrial ICD; RV, right ventricular; RVI, right ventricular implantable cardioverter-defibrillator.

The Optimizer^®^ IPG is a rechargeable device **(Figure 1)** that provides a non-excitatory stimulus to the heart when applied during the absolute refractory period of the ventricles via 2 right ventricular septal pacemaker leads, designated as “RV” (right ventricular) and “LS” (local sense), respectively. Earlier generations of the IPG incorporating CCM required an atrial lead **(Figure 2A)**; however, a dual right ventricular lead-only system is now approved (U.S. FDA October 13, 2019; Optimizer Smart™ System P180036 S003) **(Figures 2B and 2C)**. Regardless of the IPG generation, specific timing and sensing parameters need to be satisfied on a beat-to-beat basis to prevent the occurrence of CCM stimulation outside tissue refractoriness. Otherwise, capture from CCM therapy could theoretically occur and result in atrial and/or ventricular pacing, as well as arrhythmia generation. CCM therapy is programmed to occur for a limited portion of each day, typically 5, 7, or 12 h in total, in 1-h intervals dispersed evenly over a 24-h timeframe.

The current-generation Optimizer^®^ IPG contains a lithium-ion battery that is recharged by the patient, typically on a weekly basis, with the “Optimizer Mini Charger™.” The lifetime for recharging the Optimizer Smart™ battery before replacement is approximately 15 years according to the manufacturer.^9^

## Programmer

**Figure 3:**
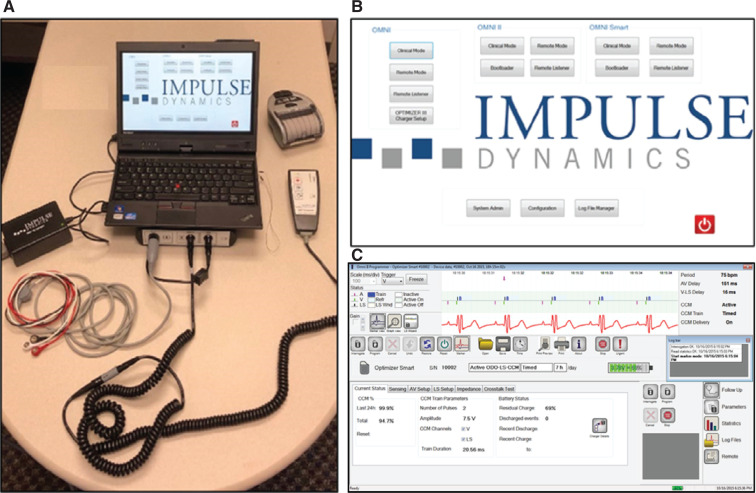
The OMNI™ II programmer system **(A)** and boot-up **(B)** and main screens **(C)**. **(B, C)** Images courtesy of Impulse Dynamics.

The OMNI™ II programmer is a battery-powered laptop used to communicate with the Optimizer^®^ IPG **(Figure 3A)**. It can be controlled in person or by a distant operator in its “Remote Mode” established via a local area network connection.^10^ To initiate in-person interrogation, the “Clinical Mode” button corresponding to the appropriate IPG is pressed on the boot-up screen to reach the main screen **(Figure 3B)**. Communication with the IPG is established when the telemetry wand is placed over the generator and the interrogation button on the wand is pushed. The programmer is recharged when plugged into a hospital-grade power outlet via a proprietary cable. In order for proper programmer operation, the charging cable needs to not be connected to the laptop when in use. If the power cable is connected to the laptop or an electrical outlet during operation, an error message will appear (“The laptop is connected to the Mains”) on the main screen to remind the user of this issue. Interrogation of the IPG is not possible in this setting.

The main screen displays the current mode of operation, IPG battery status, and real-time telemetered activity when desired in its “Marker View.” It also allows access to all programmable parameters, statistical tabulations of CCM therapy utilization, limited automatic sensing threshold testing, and the ability to generate a file saved to the laptop’s memory for later review or export **(Figure 3C)**.

Interrogation of the Optimizer^®^ IPG, depression of the markers button and then selection of the program result in the re-initiation of CCM therapy should evaluation occur when therapy is otherwise not timed to happen to allow evaluation. CCM is disabled 2–3 s after placement of a pacemaker magnet over the IPG and requires reprogramming to re-enable. The inadvertent positioning of a cardiac rhythm management (CRM) programmer wand with a magnet over the IPG will also disable CCM. This effect on the CCM IPG stands in contrast to the transient effects of placing a magnet over a pacemaker or an implantable cardioverter-defibrillator (ICD) generator whose inhibited functions are restored with its removal. The OMNI™ II programmer wand does not contain a magnet.

## Cardiac contractility modulation therapy modes and basic function

### OVO-LS-CCM mode

The default operation is to deliver CCM therapy to the heart with each sensed ventricular beat of normal rhythm when therapy is programmed to occur. In order to do so, the IPG assesses the relationship in time of the “RV” and “LS” channel sense events with respect to one another for each beat. This forms the primary mechanism for discriminating between a normal beat and an “abnormal” one, such as a premature ventricular contraction (PVC), and helps define the appropriate window in time to safely deliver a CCM pulse. CCM therapy is inhibited when it detects a PVC.

**Figure 4:**
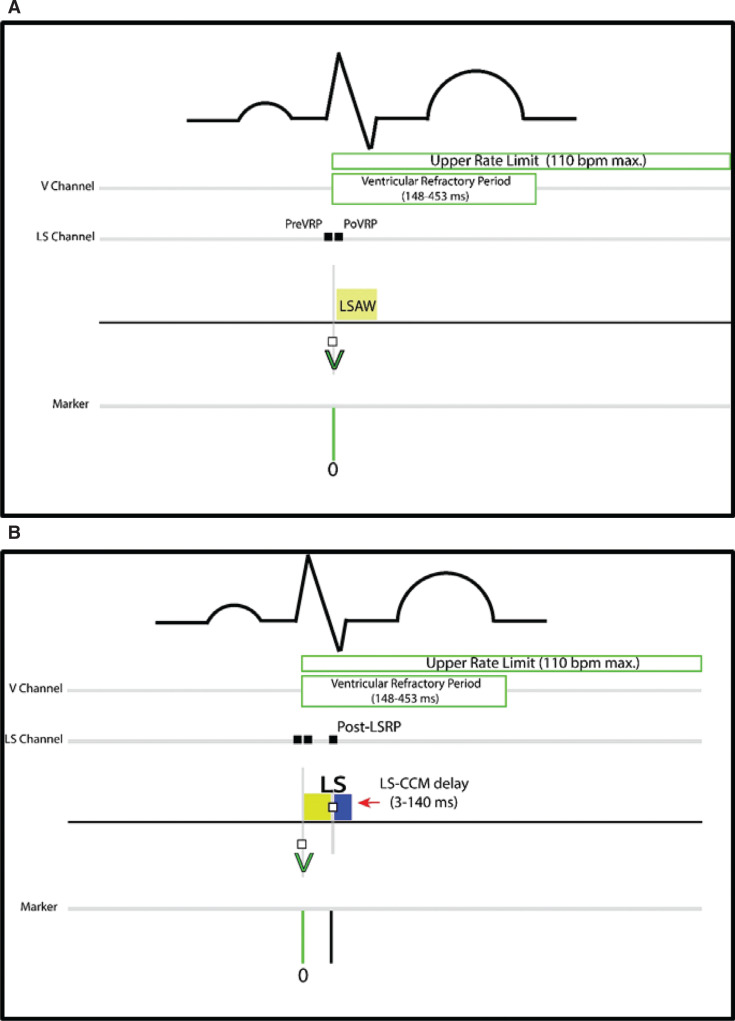

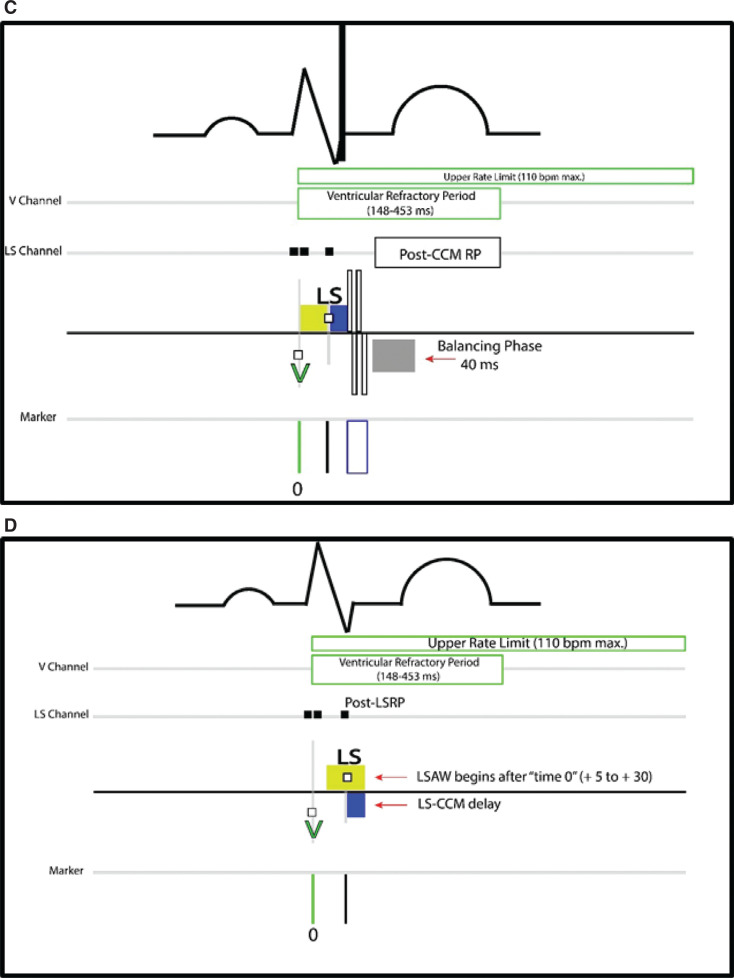

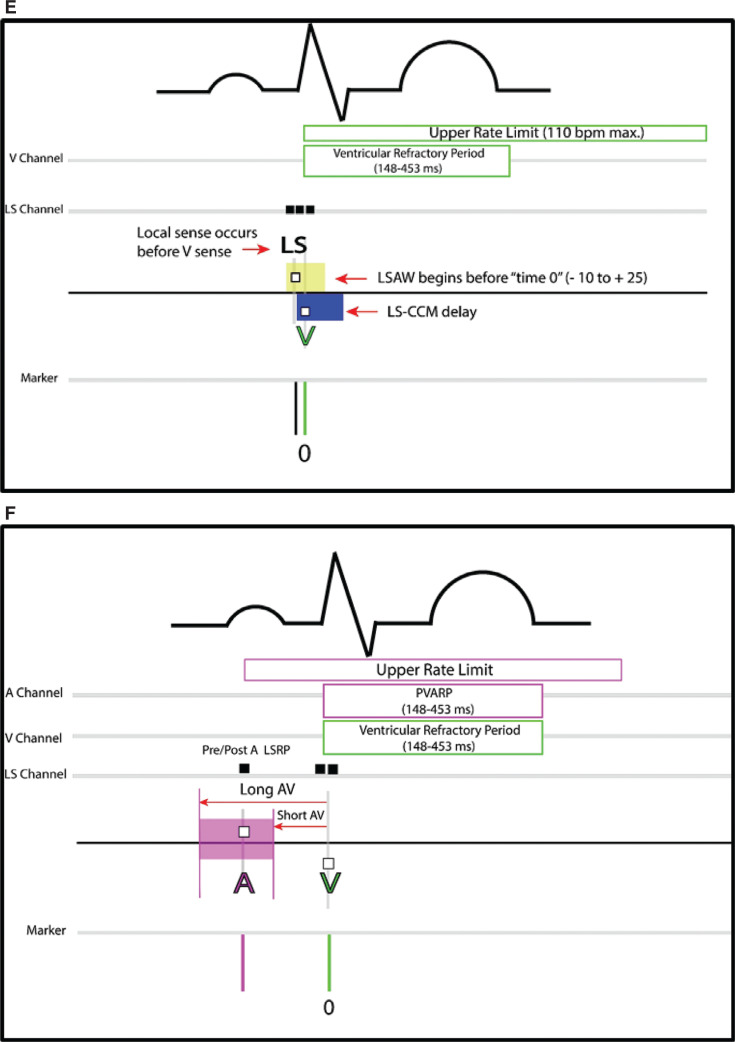
Cardiac contractility modulation (CCM) timing for OVO and ODO modes. OVO mode timing is shown in panels **A–E**. Additional criteria for ODO function are displayed in panel **F**. **A:** A V channel sense (“time zero”) begins the process and opens the local sense alert window (LSAW). **B:** An LS channel sense closes the LSAW and triggers the LS-CCM delay. **C:** CCM therapy delivers at the end of the LS-CCM delay absent an additional LS channel sense. Normal programmed timing variants may occur with an LSAW beginning a short time after **D:** or before **E:** an RV channel sense. **E:** The implantable pulse generator looks back from time zero for an LS channel sense that occurs earlier in time, but within the LSAW, to trigger an LS-CCM delay. The LS-CCM delay is adjusted in both variants **(D and E)** to time the CCM pulse properly. **F:** Atrial sensing within the long and short atrioventricular windows is required after an RV channel sense to initiate the LSAW in the ODO mode. Only following atrial sensing in this window does the process continue as seen in **A–C**. Otherwise, CCM is not delivered for that beat. *Abbreviations:* A, atrial; AV, atrioventricular; CCM, cardiac contractility modulation; IPG, implantable pulse generator; LS, local sense; LSAW, local sense alert window; LS-CCM, local sense–cardiac contractility modulation; Pre A LSRP, pre-atrial local sense refractory period; Post A LSRP, post-atrial local sense refractory period; Post-CCM RP, post-cardiac contractility modulation refractory period; PreVRP, pre-ventricular refractory period; PoLSRP, post-LS refractory period; PoVRP, post-ventricular refractory period; PVARP, post-ventricular atrial refractory period; RV and V, right ventricular.

Ventricular sensing from the CCM therapy lead determined at implant to be occurring earliest in time with each normal beat begins the process (“time zero”). This lead is plugged into the RV port of the IPG. This initiates a “local sense alert window” (LSAW) and an upper rate limit (URL) **(Figure 4A)**. The LSAW is a programmable period within which the generator looks for sensing on the therapy lead inserted into the LS port of the generator to continue the algorithm’s verification of a normal beat. The LSAW closes following a single LS channel sense event within its timeframe and initiates a programmable “LS-CCM delay” **(Figure 4B)**. A CCM biphasic impulse “train” is triggered to occur at the end of the LS-CCM delay absent an intervening additional sense on the LS lead **(Figure 4C)**. If no LS channel sense occurs during the LSAW, an LS-CCM delay is not triggered, and no CCM therapy is delivered for that beat. Following CCM delivery, a “balancing phase” of 40 ms in duration short-circuits the therapy leads to remove any residual lead polarization that might otherwise be sensed from the high-output CCM therapy. Subsequently, the IPG is alerted for RV channel sensing to restart the process for CCM delivery after clearing the ventricular refractory period (VRP) (nominal, 250 ms) and URL (nominal, 98 bpm; usually set to 110 bpm in the OVO mode). An RV channel sense event after the VRP has completed, but before the upper rate has timed out, resets the upper rate timing parameter.

The LSAW has a programmable width and a starting point. This allows a precise definition of the time range to identify the LS sensing of a normal beat with respect to the RV channel sense for any given complex. There are expected minor time variations within an individual patient regarding the relationship of RV and LS channel sensing for a normal beat **(Video 1)**. The time variation may present at implant and in follow-up as the leads mature. As such, the LSAW will not necessarily begin exactly at the moment of RV channel sensing from programming that takes this into account. The window may be programmed to begin after a short delay **(Figure 4D)**. The LSAW may also be configured to begin before an RV channel sense event **(Figure 4E)**. For this, the IPG “looks back” in time for LS sensing occurring in the window before the RV channel sense. In either programming variant, an LS-CCM delay is still triggered from a sense in the LSAW.

A PVC has a different temporal relationship between RV and LS channel sensing from that of a normal beat and also carries the potential for multiple LS sense events being detected in a single beat. This manifests as a ventricular sense above the URL with early coupling, or, with late coupling, an LS sense before the LSAW, an extra LS sense during the LS-CCM delay, or a lack of sensing during the LSAW. In all of these situations, CCM is inhibited. The FIX-HF-5C2 study reported a “new CCM delivery algorithm,” given the absence of an atrial lead, but specified no additional means for CCM inhibition from a premature beat beyond the aforementioned mechanisms.^5^

There are multiple programmable refractory periods surrounding the RV and LS sense events designed to prevent additional oversensing of a single normal beat, residual polarization occurring on the CCM ventricular therapy leads following the high-output CCM train, and the T-wave following CCM therapy delivery. The refractory periods may be adjusted to mask extra sense events from the outputs of ventricular or His-bundle pacing. Ventricular pacemaker spikes and captured beats may display disparate/multiple RV and LS channel sensing in time like a native PVC. This would tend to inhibit CCM all of the time without attention to alternate ventricular sensitivity or refractory period programming.

### ODO-LS-CCM mode

In contrast to the OVO mode, the ODO mode affords a higher maximum upper rate CCM therapy capability (179 vs. 110 bpm), requires the insertion of an atrial pacemaker lead, and introduces additional criteria that need to be satisfied before a CCM pulse train may be delivered for a given beat. RV channel sensing initiates the process as in the OVO mode. In order for an LSAW to be opened, the IPG needs to have detected an atrial sense within a specified window preceding the RV channel sense **(Figure 4F)**. The IPG looks back in time within a window defined by 2 programmable parameters: the “short AV” and “long AV.” If no atrial sensing occurs within this timeframe, CCM therapy is not delivered for the beat. If atrial sensing occurs within this atrioventricular (AV) window, the process of events then proceeds as in the OVO mode.

CCM inhibition is not limited to detecting a PVC. Sensing >1 LS channel event for a single beat, no LSAW LS sensing, lacking atrial sensing in the ODO mode, and sensing above the URL will also result in inhibition conditions. Exposure to external magnetic interference, while not described in any prior studies in CCM, would be expected to result in an inhibition condition due to extra sensed events.

### Response to inhibition

**Figure 5:**
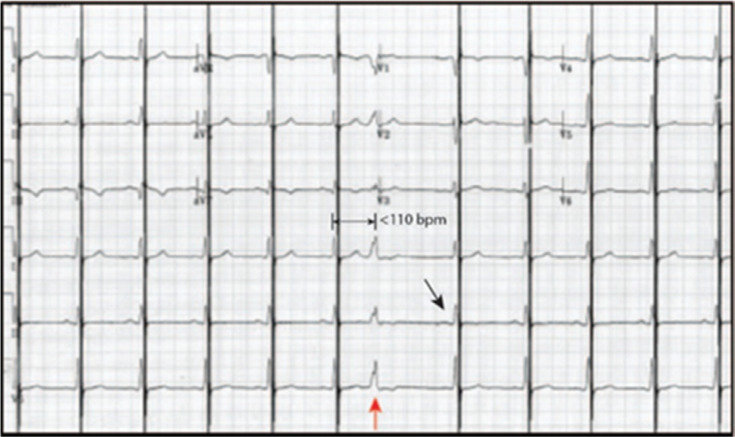
Twelve-lead electrocardiogram with cardiac contractility modulation (CCM) in the OVO mode. The implantable pulse generator recognizes the seventh beat as a premature ventricular contraction under the upper rate limit of 110 bpm (red arrow), inhibiting CCM therapy. The post-inhibition response is programmed to “0” indicated by CCM being delivered with the next atrial paced and conducted beat (black arrow).

When an inhibition condition occurs, a programmed response of continued inhibition from 1–15 of the following beats is triggered in the current-generation IPG (see example in **Figure 5**). Each subsequent beat happening in this period that would otherwise also fulfill an inhibition criterion by itself continues to reset this response. This function is carried over from earlier generations of the Optimizer^®^ IPG. Its purpose is to ensure a lack of pro-arrhythmia from the therapy. This parameter is commonly programmed to “0” additional beats as completed trials of CCM have not demonstrated pro-arrhythmia from the therapy.^5–8^ Earlier generations of the Optimizer^®^ IPG will not allow the response to be <2 beats. As such, this has the potential to limit the therapy delivery volume necessary to provoke a response and may require an increase in h/day of therapy delivery to overcome.

### OOO mode

The OOO mode is also classified as the “standby” mode. No events are sensed and CCM therapy is not delivered.

### Cardiac contractility modulation off status

As mentioned earlier, placement of a pacemaker magnet over the IPG disables CCM therapy delivery by placing it into a “permanent off” position or the more commonly described “magnet mode” state. Despite the lack of therapy delivery, the device continues to sense and statistically classify events in its counters. Only through reprogramming can this status be changed to another mode of function. The other off status is called the “down mode” and is automatically assumed by the IPG in the unlikely circumstance of detecting significant inconsistency in its logic circuits. This situation occurs when either of the 2 IPG microprocessors detects a problem with the flow of information in the other. No CCM therapy is delivered, and sensing does not occur. This state can only be changed by a reset via the OMNI™ II programmer.^10^

### Implant considerations

**Figure 6:**
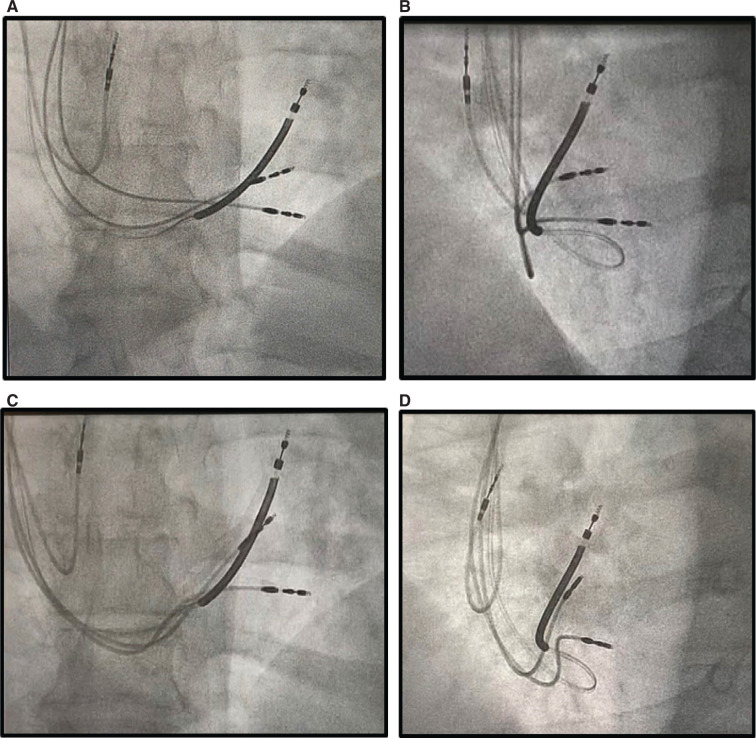
Anteroposterior and 40° left anterior oblique fluoroscopic ventricular lead positions during cardiac contractility modulation (CCM) implant. The implantable cardioverter-defibrillator lead was previously implanted high in the outflow tract. The initial CCM ventricular lead positions show dyssynchronous movement, and the tips move apart with beating **(A and B)**. Repositioning the ventricular leads results in tandem movement without tip separation **(C and D)**. The superior CCM lead is felt to be appropriately distanced from the base of the heart. See **Videos 2A–2D** for more information.

In order to facilitate an optimally functioning CCM system, proper placement of 2 septal ventricular pacemaker leads at the minimum needs to occur, taking into consideration the Heart Rhythm Society/European Society guidelines regarding the recommended maximum number of indwelling endovascular leads.^11,12^ A ventricular basal position is not recommended to prevent the likelihood of atrial capture, oversensing of far-field atrial signals by a ventricular lead, or far-field sensing of the CCM pulse by an existing CRM device. Spatial separation of ≥2 cm from each other and distance from other CRM device elements will minimize physical and electrical interactions between them. Steep oblique fluoroscopic views help to confirm a septal location, along with the observation that the CCM leads move together with each beat (ie, they are attached to the same piece of tissue). Absent pre-existing septal dyskinesis leads that separate farther apart and/or do not move in tandem with beating imply that one of them is attached elsewhere **(Figures 6A–6D and Video 1)**. A most common position is in the “crux” between the septum and the anterior free wall of the right ventricle. This becomes clinically relevant not because an off-septum position of one of the leads necessarily prevents a response to CCM therapy, but rather there is the potential for chest wall discomfort due to the high-output CCM therapy with anterior positioning. The mechanism is presumed to be from the stimulation of intercostal muscles due to an anterior location.^13^ It may be assessed at implant by allowing some arousal and querying the patient for the presence of chest discomfort with CCM delivery. As well, focal contraction of the chest wall may be observed in our experience. This provides another opportunity to determine the stability and feasibility of the lead positions before the procedure is completed. Otherwise, intractable chest discomfort post-implant may subsequently require an offending lead to have its output amplitude turned down, be turned off for CCM delivery, or repositioned if felt necessary. Of note, there are data supporting a response to single-lead CCM therapy delivery.^14^

Ventricular pacing thresholds at CCM implant are determined primarily to confirm lead stability but are not necessarily a determining factor of a functionally appropriate lead position, as pacing capture is not a feature of CCM therapy. Rather, adequate sensing capabilities need to be obtained, similar to those in a traditional CRM implant, to allow appropriate interval triggering and inhibition when needed.

After achieving satisfactory ventricular lead positions, the comparative timing for sensing a normal beat is evaluated by attaching 2 sets of alligator clips to these leads. The ventricular lead consistently sensing a normal beat earlier in time is then inserted into the RV, and the other is inserted into the LS port of the IPG. The parameters for the LSAW and LS-CCM delay are then adjusted accordingly to time the CCM pulse delivery appropriately.

## Device–device interaction

### Implantable cardioverter-defibrillator and cardiac contractility modulation

Many patients undergoing implantation of a CCM system have a pre-existing CRM device. Of particular importance are those with an ICD. ICDs may potentially sense the CCM pulse train in addition to native R-waves, which can lead to inappropriate tachycardia detection under the right circumstances from double counting (think sinus tachycardia under the URL of the CCM IPG). The likelihood of this occurring clinically and causing inappropriate ICD tachycardia detection is least likely to occur in the OVO mode given the maximum URL of 110 bpm for CCM. Regardless, to minimize this likelihood, a “crosstalk test” is performed at implant before the IPG pocket is closed. This entails first turning off ICD tachycardia detection, programming maximum ICD ventricular sensitivity, selecting the “Crosstalk Test” tab on the OMNI™ II programmer, and monitoring the ICD marker channels on its programmer to see if the CCM signal is sensed. The LS-CCM delay automatically defaults to 85 ms for the test. If the CCM pulse train is detected by the ICD (in other words, double-counted), programming a decreased ICD ventricular sensitivity, an increased ventricular blanking period in the ICD, and/or shortening the LS-CCM delay is expected to mask sensing of the CCM signal. Should that not resolve CCM train detection, repositioning of the therapy lead closest to the defibrillator lead is likely to settle the issue. Alternatively, a recently reported case of ICD tachycardia double counting was resolved by turning off therapy in one of the CCM leads.^15^ Following the crosstalk test, the programmed parameters of the LSAW and LS-CCM delay are then reconfirmed to deliver CCM into the appropriate window in time. Induction of ventricular fibrillation (VF) with a co-existing ICD, while not necessary, is frequently performed to ensure appropriate sensing and inhibition of CCM by the IPG and detection and treatment of VF by the ICD.

**Figure 7:**
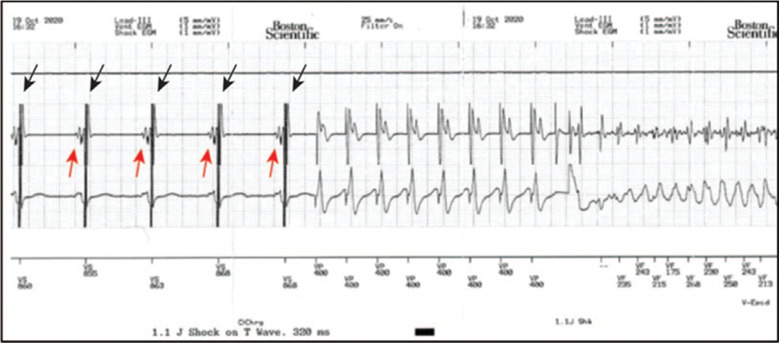
Native signal undersensing from cardiac contractility modulation (CCM) before ventricular fibrillation (VF) induction. The single-chamber defibrillator has a non-programmable ventricular blanking period. The red arrows indicate the undersensed native signals on the near-field ventricular electrogram. The automatic gain function results in only CCM signal sensing by the defibrillator initially (black arrows). CCM therapy is immediately inhibited with induction and remains inhibited during VF. VF detection is not delayed nor is VF undersensed.

Undersensing of the native R-wave by an ICD can occur in normal rhythm when CCM therapy falls outside the ICD ventricular blanking period. The native R-wave is subsequently skipped because ICD sensing of the large-amplitude CCM pulse train forces the electrogram signal to be either gained down or the ventricular sensitivity level to be elevated high enough before stepping/decaying down such that the ICD does not see the comparatively smaller native signal on the next beat **(Figure 7)**. The ICD blanking period may not be programmable to account for this. Turning off therapy in one of the CCM leads does not resolve the issue in our experience. Changing the decay or step-down for sensing has not been reported to alter this phenomenon either. Clinically to date, this condition has not delayed or prevented tachycardia detection and treatment.

Automatic native R-wave template acquisition in an ICD should be turned off after CCM insertion to prevent a beat with CCM therapy being used for tachycardia morphology discrimination.

### Pacemaker function/native ventricular conduction delay and cardiac contractility modulation

The output(s) of ventricular or His-bundle pacing with evoked response and ventricular conduction delay potentiate >1 signal sense in a short span of time on the LS channel of the CCM IPG. An LS channel sense event outside of the LSAW, including during the LS-CCM delay, results in a CCM inhibition condition. Theoretically, decreasing the LS channel sensitivity might allow elimination of extra sense events. Another option is to program the pre-/post-RV channel LS refractory period to eliminate an early LS signal, then program the LSAW and post-LS channel LS refractory period to sense only a single later LS signal component within this window. Such timing adjustments should decrease the likelihood of inhibition during the LS-CCM delay. A resultant effect of such programming might be the clinically benign delivery of CCM therapy timed into the absolute refractory period of a late-coupled PVC under the URL in the OVO mode. On the other hand, Tint et al. described turning off right ventricular pacing in a biventricular device as a means for preventing CCM inhibition.^15^ Of note, CCM is not currently labeled for use with biventricular pacing. As such, turning off left ventricular pacing might also eliminate extra LS channel sense events.

Pacemaker sensing of the CCM signal outside of its VRP is not expected to occur and inhibit the ability to sense a subsequent native R-wave signal (as seen in **Figure 7**). This is because of the fixed sensing level functionality of a pacemaker.^16,17^

### Other implant considerations

Additional programming options at implant include determining the number of pulses per delivery and daily hours of CCM therapy. The number of pulses per delivery is programmable from 1–3 but typically kept at an out-of-the-box count of 2. Five hours of daily therapy was typical in the FIX-HF study^6,18^ and ≥7 h was typical in European studies.^7,19^ CCM is currently labeled for as high as 7 h/day in the United States.

### Follow-up considerations

The follow-up workflow for the Optimizer^®^ IPG is significantly different from that for a traditional CRM device. This is primarily because threshold measurements play a smaller role, and determination of CCM delivery efficiency represents a dominant feature. As its follow-up schedules are typically similar to those of a traditional CRM device, interrogation of a co-existing defibrillator or pacemaker may occur at the same visit. This and remote monitoring help ensure the absence of crosstalk tachycardia detections.

After IPG interrogation, the main programmer screen displays the “current status” tab and the CCM percent delivery **(Figure 3C)**. It is automatically calculated for the last 24 h and for the generator since the last counter reset. This is derived by taking the number of CCM trains delivered and dividing by the number of eligible beats for CCM in that timeframe. The IPG does not automatically report the value since the last follow-up at present. In order to generate this information, a reset of the counters at each visit is required. This is strongly suggested as a means for optimizing follow-up troubleshooting. A minimum target for percent CCM delivery can be programmed into the IPG on its alarm panel as determined by the clinician. If the target is subsequently not met, a numeric message code will appear on the Optimizer Mini Charger™ to alert the patient the next time the IPG is charged. It is analogous to the percent biventricular pacing sought in a cardiac resynchronization device. The target for percent CCM delivery was 70% for a 5-h/day scheme in the FIX-HF4 study. This was predicted to result in approximately 15,120 CCM trains delivered per day in order to provoke a response and was replicated in the FIX-HF 5C2 study.^5,7^ The actual percent CCM delivery, however, has not been reported to date in any of the major trials in CCM to otherwise define a specific target.^5–8^ Regardless, these data and clinical assessment of response dictate the process of what occurs in the follow-up evaluation. In other words:

1. Is the percent of CCM delivery adequate?
2. If not, what are the cause(s) of therapy inhibition?
3. Can reprogramming timing intervals, sensitivities, or altering native rhythm management increase the CCM delivery percent?
4. If not, does the number of hours of daily CCM therapy delivery need to be increased to affect the overall volume of delivery?

Conditions causing therapy inhibition are statistically tabulated and stored in the IPG from the last reset. Categorization of inhibition facilitates troubleshooting by suggesting a potential path for maximizing CCM therapy delivery when it is deemed suboptimal. When evaluating the modes of inhibition, it is important to understand that a singular issue can end up in different categories depending on the time of occurrence and programmed active CCM mode. For example, an actual PVC above the URL appears in the “ventricular tachycardia” category in the OVO mode. The same PVC is categorized as a “premature ventricular contraction” in the ODO mode because it is not preceded by an atrial sense, or as an “LS out of alert” in the OVO mode with later coupling. Conversely, a single category of inhibition can have multiple different causes. Loss of atrial sensing in the ODO mode causes a normal beat under the URL, as well as a PVC, to appear in the “premature ventricular contraction” category. Monitoring the IPG with the “marker” icon selected and a surface electrocardiogram (ECG) lead connected enhances the ability to further differentiate and identify the ultimate cause(s) within each category. Management strategy will be different depending on the actual cause of inhibition.

**Figure 8:**
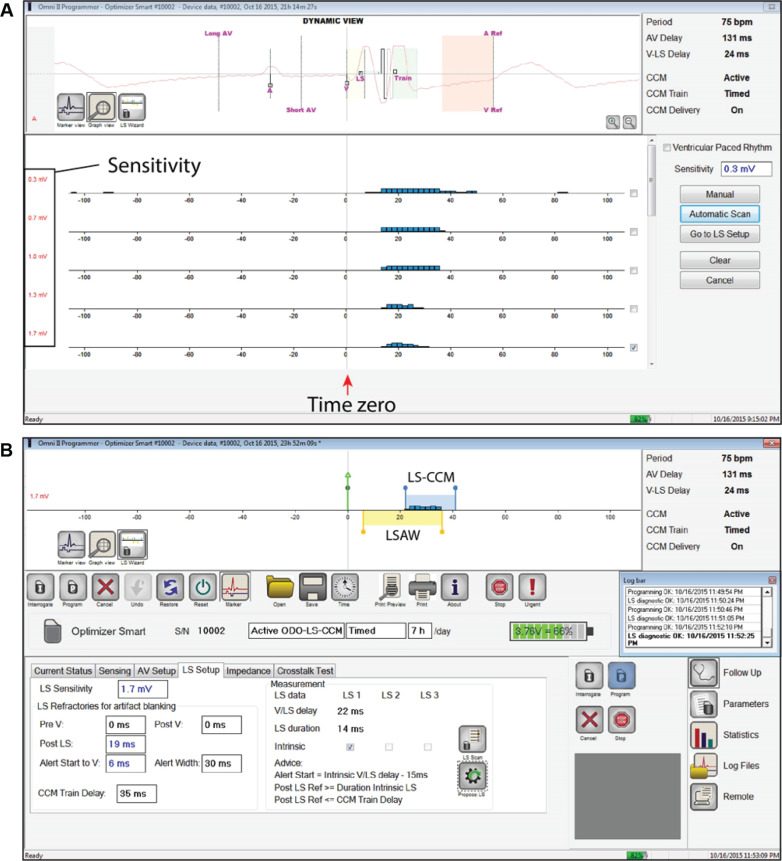
LS scan test and LS setup on the OMNI™ II Programmer. **A:** LS channel signals measured with repeat sampling at 0.3–1.7 mV (blue histograms) and with respect to right ventricular (RV) channel sensing (time zero) after the “Automatic Scan” button is selected. More sensitive settings detect signals that are not specific for the near-field LS pacemaker lead and sense the single local beat multiple times. The scan suggests an LS channel sensitivity of 1.7 mV for programming. **B:** The display when “Go to LS setup” is chosen. The LS channel sensitivity, refractory periods, local sense alert window (LSAW), and LS-CCM delay are programmed here. The LSAW begins +6 ms after V channel sensing and extends out to +36 ms from time zero. The LSAW (yellow) and LS-CCM delay (blue) windows are shown in a linear timeline at the top of the panel. Images courtesy of Impulse Dynamics.

Additional values assessed at follow-up typically include battery status, lead impedance, and sensing. The “LS scan function” on the programmer displays both LS sensing and occurrence in time with respect to the event that initiates the CCM algorithm, RV channel sensing **(Figure 8)**. This function facilitates determining the LS channel sensing “threshold,” programming sensitivity level on the LS channel, and a re-configuration of the LSAW if needed. It also provides a visual display of multiple senses on the LS channel for a single beat if they occur. Otherwise, the atrial sensing threshold has to be determined manually by reprogramming the respective sensitivity level, while monitoring marker channels for loss of sensing. Pacing thresholds cannot be determined post-implant through the IPG.

Other issues may need to be addressed in follow-up as dictated by automatic numeric error messages that appear on the IPG charger **(Table 1)**. Some refer to potential lead problems that are familiar for troubleshooting to any CRM device specialist. Lead fracture, insulation break, and dislodgement were the most frequently cited complications in the FIX-HF-5 and FIX-HF-5C studies.^6,8^

Manufacturer recommendations regarding the exposure of the IPG to environmental conditions that might interfere with the IPG function are detailed in the pulse generator technical manual.^9^ More recently (January 19, 2021), the U.S. FDA granted conditional approval for the use of cardiac magnetic resonance imaging with CCM.

A review of the IPG recharging process with the patient during follow-up is also beneficial in our experience. This commonly addresses and reassures patients’ concerns over the length of time taken to recharge the IPG.

Queries regarding chest wall discomfort are standard in our clinic. Potential reprogramming options, should this be of significance, include reducing the CCM pulse amplitude, turning off therapy in a lead, or ultimately repositioning a lead if needed. Out-of-the-box characteristics for a biphasic CCM train comprise an amplitude of 7.5 V with 2 pulses over a 20.56-ms duration.^10^

Finally, remote IPG monitoring capabilities are not currently offered, but are expected to be available within the next calendar year (Impulse Dynamics, personal communication).

## Discussion

Newer implantable device technologies, including CCM, that were not previously available for the management of heart failure in patients not indicated for biventricular pacing therapy now exist.^5–8,20^ As a natural consequence, a demand for familiarity with the technology has arisen in order to facilitate appropriate patient management. This demand will only become more magnified with further expansion of the technology. Initiation of the Assessment of CCM in Heart Failure with Higher Ejection Fraction (AIM HIGHer) study of CCM in patients with an ejection fraction of 40%–60% has started (NCT05064709), and the “Integra-D” study of CCM encompassed in an ICD, which is anticipated to start enrolling later this year (FDA protocol approval pending), will also add to prior trial experience. CCM use in failed biventricular pacing response has also been described by Nagele et al.^13^ and Kuschyk et al.^21^ Results from these works have the potential to expand CCM indications.

This review is provided to address the technical knowledge deficit for CCM as the literature is lacking on the topic. A simple replication of technical manuals is insufficient for this purpose due to the absence of clinical context and complexity of CCM function. Here, basic timing function is visually described in a simple fashion. Troubleshooting problems are summarized, including potential pacemaker lead issues that are already familiar to any device specialist. Solutions are proposed, but we do not purport to cover all eventualities that may occur. A clarification from completed trials regarding a CCM train delivery target for patient response is paramount and will simplify the follow-up strategy. Future generations of CCM will also be integrated into traditional CRM devices. It would be reasonable to anticipate a revised methodology to simplify beat discrimination that includes morphology detection. Enhanced capabilities for CCM, including closed loop management of decreased CCM delivery and LSAW configuration, would not be unreasonable to expect as well. As a result, new opportunities for CCM technical education expanding on this foundational review will occur.

Becoming familiar with the OMNI™ II programmer is necessary for currently understanding CCM. One cannot simply extrapolate experience with traditional CRM programmers to CCM, as many of the programmable variables do not overlap. Finding the necessary parameters on the programmer and developing a follow-up best practice workflow take experience and time. A greater description of CCM programmer function is beyond the scope of this review. It is expected that a revised programmer with improved user-friendliness and enhanced follow-up automation is forthcoming.

Another issue deserving mention is the proprietary designation for the active CCM modes OVO-LS-CCM and ODO-LS-CCM. At first glance, for the device specialist “purist,” this would seem to be inconsistent with the revised North American Society of Pacing and Electrophysiology/British Pacing and Electrophysiology Group pacemaker code.^22^ With CCM, ventricular therapy is triggered to occur after a single ventricular LS channel sense—that is, VVT function—absent a subsequent LS channel sense outside of LS refractory periods within the LS-CCM delay. This triggering functionality is familiar to those old enough to have used a temporary VVT pacing mode for diagnostic sensing purposes and can look remarkably similar to CCM on the surface ECG.^23^ What seems to be missing is a multilead sensing designation in the pacemaker code (position 6?). This will allow the ability to describe inhibition of a ventricular CCM output after an initial trigger. Thus, the active modes describing solely CCM appear as VVDOVV and VDDOVV instead of OVO-LS-CCM and ODO-LS-CCM, respectively. What becomes a bit more cumbersome is the potential mode designations in future single devices encompassing both pacemaker capture and CCM non-capture functions. It may come to pass that 2 separate mode designations following the code and reflecting this dichotomy will be necessary. This separate discussion may deserve further elaboration at a future date.

## Conclusions

Understanding CCM therapy is within the capability of the motivated individual who seeks to master its functionality. As a result, the potential for both improved patient management in those already implanted with this therapy and enhanced expansion to those eligible but not yet treated with CCM would seem more likely.

## Figures and Tables

**Video 1:** Real-time marker channels in “Dynamic View.” The video shows beat-to-beat time variation of LS sensing and cardiac contractility modulation train delivery with respect to RV channel sensing (time zero). Note the local sense alert window begins shortly after the RV channel sense.

**Video 2A:** Cine video of **Figure 6A**.

**Videos 2B:** Cine video of **Figure 6B**.

**Videos 2C:** Cine video of **Figure 6C**.

**Videos 2D:** Cine video of **Figure 6D**.

**Table 1:** Charger Error Codes

Numeric Code	Code Description	Source	IPG Rechargeable? (Y/N)	Suggested Action
0	IPG deactivated	EMI or internal software conflict	N	Prompt follow-up for assessment and reactivation
1	Lead impedance change	Normal post-implant change vs. lead malfunction	Y	Prompt follow-up if message persistently recurring
2	CCM inactive	Exposure to strong magnetic field or high current condition with low impedance	Y	Prompt follow-up for impedance evaluation
3	CCM delivery not programmed	CCM turned off or in “standby”	Y	Prompt follow-up for reactivation
4	% CCM therapy low	Sensing changes, arrhythmia, or lead malfunction	Y	Prompt follow-up for arrhythmia management, mode, sensing, impedance, and/or detection window adjustment
5	Optimizer Smart™ **temperature issue**	Febrile illness or abnormal IPG/charger interaction	Not suggested	Pause charging, prompt follow-up, physical exam
6	**Internal charger fault**	Charger malfunction	N	Prompt follow-up for charger replacement
7	IPG not Optimizer Smart™ **device**	Wrong-generation Optimizer charger	N	Re-educate appropriately matched charger use
8	Battery depleted	Battery discharged completely or has operation interference	Not suggested	Pause charging, prompt follow-up

*Abbreviations:* CCM, cardiac contractility modulation; EMI, electromagnetic interference; IPG, internal pulse generator; N, no; Y, yes.
